# PET/CT鉴别肺癌中胸腔积液性质的临床价值

**DOI:** 10.3779/j.issn.1009-3419.2012.11.08

**Published:** 2012-11-20

**Authors:** 日强 廖, 学宁 杨, 思云 王, 清 周, 强 聂, 文昭 钟, 嵩 董, 一龙 吴

**Affiliations:** 1 510080 广州，广东省人民医院肿瘤中心肺科，广东省肺癌研究所，广东省医学科学院 Department of Pulmonary Oncology, Cancer Center, Guangdong General Hospital, Guangdong Lung Cancer Research Institute, Guangdong Academy of Medical Sciences, Guangzhou 510080, China; 2 510080 广州，广东省人民医院肿瘤中心核医学科，广东省肺癌研究所，广东省医学科学院 Department of Nuclear Medicine, Guangdong General Hospital, Guangdong Lung Cancer Research Institute, Guangdong Academy of Medical Sciences, Guangzhou 510080, China

**Keywords:** PET/CT, 肺肿瘤, 恶性胸腔积液, 诊断, PET/CT, Lung neoplasms, Malignant pleural effusion, Diagnosis

## Abstract

**背景与目的:**

胸腔积液是肺癌患者常见的临床表现，鉴别胸水的性质有重要意义。正电子发射断层扫描/计算机体层摄影（positron emission tomography/computed tomography, PET/CT）是鉴别肺部良恶性肿瘤和纵隔淋巴结分期的重要诊断方法之一。本研究旨在探讨PET/CT鉴别肺癌中胸腔积液性质的临床价值。

**方法:**

回顾性分析合并胸腔积液的病理确诊的肺癌病例，以病理或临床随访为最后诊断标准，计算PET/CT诊断肺癌恶性胸腔积液的敏感性、特异性、阴性预测值、阳性预测值和准确率。

**结果:**

33例肺癌患者符合条件纳入分析。PET/CT诊断肺癌恶性胸腔积液的敏感性、特异性、阳性预测值、阴性预测值和准确率分别为81.5%、83.3%、95.7%、50.0%和81.8%。

**结论:**

PET/CT对鉴别肺癌中胸腔积液的性质具有重要作用，假阳性率低，对PET/CT阴性的胸腔积液，需有创检查确认。

## Abstract (en)

**Background and objective:**

Pleural effusions, a common feature among patients with lung cancer, should be differentiated into benign or malignant pleural effusions. F-18 fluorodeoxyglucose positron emission tomography/computed tomography (PET/CT) is one of accurate diagnostic tool for differentiating benign from malignant disease and for mediastinal lymph node staging. We explored the clinical role of PET/CT for differentiating malignant pleural effusion from benign pleural effusion among patients with lung cancer.

**Methods:**

We retrospectively reviewed the data from all patients with lung cancer and pleural effusion who underwent F-18 FDG PET/CT. The PET/CT results were compared with the pathologic diagnosis or clinical follow-up. The sensitivity, specificity, positive predictive value, negative predictive value, and accuracy of PET/CT for detecting malignant pleural metastases were calculated.

**Results:**

A total of 33 patients were enrolled. The sensitivity, specificity, positive predictive value, negative predictive value, and accuracy of PET/CT were 81.5%, 83.3%, 95.7%, 50.0%, and 81.8%, respectively, for detecting malignant pleural effusions lung cancer patients.

**Conclusion:**

Our results suggest that F-18 FDG PET/CT can play a significant role in diagnosing lung cancer patients with pleural diffusions, with low false-positive rate. However, negative findings under PET/CT should be confirmed through further tests.

胸腔积液（胸水）是肺癌患者常见的临床表现，但是并非所有的胸腔积液都是恶性，鉴别胸水的性质对明确分期和制定治疗方案具有重要意义。影像学检查如计算机体层摄影（computed tomography, CT）、磁共振成像（magnetic resonance imaging, MRI）等，诊断恶性胸腔积液的敏感性高，但特异性和准确率不高。胸腔穿刺术和胸膜活检术是最常用的有创检查，其中胸腔镜下胸膜活检术的诊断率最高，但需要特殊的设备，难以在临床中常规使用。

正电子发射断层扫描/计算机体层摄影（positron emission tomography/computed tomography, PET/CT）能鉴别肺部结节的性质，与CT相比，其纵隔分期的敏感性、特异性更高，而且能减少无效开胸率。目前，有关PET/CT诊肺癌胸腔积液性质的研究^[1]^较少，国内未见较大样本的报道。本研究旨在回顾性分析广东省人民医院收治的肺癌合并胸腔积液的患者并行PET/CT检查的资料，探讨PET/CT在肺癌患者中鉴别其胸腔积液性质的价值。

## 1 材料与方法

### 1.1 入组条件

广东省人民医院肿瘤中心2009年1月-2011年6月病理确诊肺癌且满足以下条件的病例：①合并胸腔积液伴或不伴胸膜结节；②在我院行PET/CT检查；③有胸水细胞学或胸膜组织学诊断者。排除标准包括：①合并其它恶性肿瘤；②只行PET检查或外院行PET/CT检查；③无胸水细胞学或胸膜组织学诊断；④只行1次胸腔穿刺细胞学检查，且未获得明确诊断者。胸水判定标准：①少量胸水：CT可见胸水，但胸部立位片肋膈角未变钝；②中等量胸水：肋膈角变钝，胸水低于第2前肋；③大量胸水：胸部立位片胸水超过第2前肋。血性胸水定义为胸水呈肉眼红色。

### 1.2 PET/CT检查流程及诊断标准

本研究中，所有的PET/CT检查都为同一部机器，按照统一的步骤进行操作，从而最大限度地减少机器和操作导致的误差。步骤如下：所有检查者空腹4 h-6 h以上，测空腹血糖 < 8 mmol/L，注射药物18氟代脱氧葡萄糖（18F-FDG），注射剂量0.2 mCi/kg。注药后检查者安静避光平卧60 min进行检查。PET/CT设备型号为Biograph16，Siemens，German。先行体部CTAC（computed tomography attenuation correction）扫描，确定扫描范围（双侧股骨上段至颅底），管电压120 kV，管电流50 mAs。再行体部PET扫描，3D采集模式，2 min/床位，每位患者约5个-7个床位。后行头颅CTAC扫描，管电压120 kV，管电流200 mAs。再行头颅PET扫描，3D采集模式，5 min/床位，1个床位。PET/CT图像重建完毕，利用Wizard工作站MSV软件进行融合处理。如果符合以下条件之一者诊断为阳性：①胸膜SUVmax≥2.5；②胸膜多发结节并代谢增高者（即使SUVmax < 2.5）。

恶性胸腔积液的诊断标准：①胸水细胞学发现癌细胞；②胸膜活检发现癌细胞。如果胸水细胞学或胸膜活检未发现癌细胞，需随访1个月没有胸水复发方诊断为良性胸腔积液（因为恶性胸水只是行胸腔穿刺术，1个月后几乎都复发^[2]^），以上述标准为胸腔积液良恶性的最终诊断标准，满足以下条件之一者均诊断为恶性胸腔积液，计算PET/CT诊断肺癌恶性胸腔积液的敏感性、特异性、阳性预测值、阴性预测值和准确率。

### 1.3 统计分析

应用SPSS 13.0软件计算PET/CT诊断肺癌并恶性胸腔积液的敏感性、特异性、阳性预测值、阴性预测值和准确率。

## 2 结果

2009年1月-2011年7月间共33例肺癌患者符合上述条件纳入分析（表 1），其中30例为初治，3例复治。33例患者中，27例最后诊断为恶性胸腔积液，另外6例诊断为良性胸腔积液，其中2例患者接受肺癌完全切除术，术中未见恶性胸腔积液和胸膜转移，术后胸腔冲洗液也未发现癌细胞，最后病理分期为Ⅱ期，其余4例患者1个月后胸水未复发（1例Ⅱ期患者接受放疗，1例Ⅲ期小细胞肺癌患者接受根治性化放疗，1例Ⅳ期患者接受化疗，1例Ⅳ期患者只行胸腔穿刺术，未接受化疗或靶向治疗）。

**1:** 入组患者的特征和胸腔积液的诊断 Characteristics and final diagnosis in patients enrolled in the study

Variable	*n*	Proportion (%)
Gender		
Man	24	72.7
Female	9	27.3
Age (yrs)		
Mean±SD	60.7±12.3	-
Range	37-85	-
Pathology		
Adenocarcinoma	27	81.8
Squamous cell carcinoma	2	6.1
Small cell lung cancer	4	12.1
Pleural effusion		
Large	7	21.2
Moderate	20	60.6
Small	6	18.2
Blood	20	60.6
Non-blood	13	39.4
Malignant	27	81.8
Benign	6	18.2
Stage		
Ⅱ-Ⅲ	4	12.1
Ⅳ (M1a)	13	39.4
Ⅳ (M1b)	16	48.5

PET/CT诊断23例肺癌合并恶性胸腔积液（表 2），22例为真阳性（图 1），1例通过内科胸腔镜诊断为结核性胸膜炎，抗结核治疗后接受完全性肺癌切除术，术后病理分期为Ⅱ期，判断为PET/CT假阳性；PET/CT诊断10例良性胸腔积液，其中5例假阴性。PET/CT诊断肺癌恶性胸腔积液的敏感性、特异性、阳性预测值、阴性预测值和准确率分别为81.5%、83.3%、95.7%、50.0%和81.8%。如果以PET/CT的SUVmax≥2.5作为诊断恶性胸腔积液的标准，其敏感性下降为59.3%，而阴性预测值更低至31.3%。

**2:** 33例合并胸腔积液肺癌患者PET-CT和根据SUVmax的诊断结果 Diagnostic results of PET-CT and SUVmax in pleural effusion in 33 patients with lung cancer

Final diagnosis	PET-CT			PET-CT（SUVmax）		Total
	（+）	（-）		（+）	（-）	
（+）	22	5		16	11	27
（-）	1	5		1	5	6
Total	23	10		17	16	33

**1:**
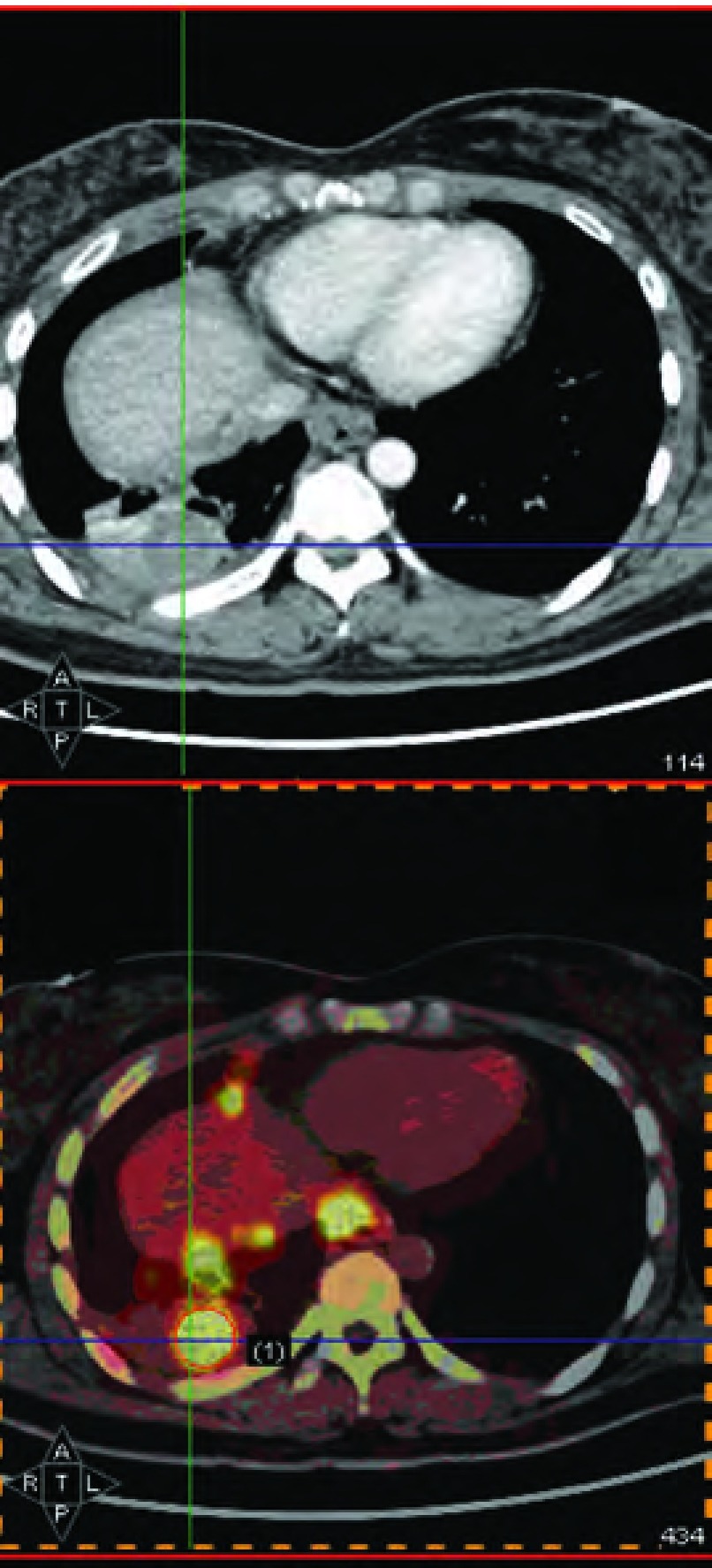
PET/CT显示右侧胸腔积液，胸膜增厚，SUVmax升高，诊断肺癌合并恶性胸腔积液。 PET/CT shows right-side pleural effusion and reveals nodular thickening increased uptake in parietal pleura.

## 3 讨论

本回顾性研究显示，PET/CT是敏感性和特异性较高的诊断肺癌合并恶性胸腔积液的无创方法，其敏感性、特异性和准确率为81.5%、83.3%和81.8%，阳性预测值95.7%，而阴性预测值只有50.0%。胸腔积液是肺癌患者常见的临床表现，在整个疾病发展过程中，10%-50%的患者将出现恶性胸水^[3]^。但胸腔积液并非都是恶性，例如肺不张、阻塞性肺炎和其它炎症性病变，如结核等也可能引起胸水。因此鉴别胸腔积液的性质对明确分期和制定治疗方案具有重要意义。CT以胸膜结节样增厚或壁层胸膜增厚超过1 cm作为诊断恶性胸腔积液的标准，敏感性88%-100%，特异性22%-56%^[4-6]^。MRI对软组织的分辨率高于CT，能更清楚地显示肿瘤是否侵犯胸壁或膈肌，而且能辨别胸膜形态学的变化，因此诊断恶性胸膜病变的敏感性和特异性与CT相似或更高^[7]^。但由于MRI对肺实质的显示不如增强CT，因此MRI不作为胸腔积液的常规检查，而只用于复杂病例的鉴别。胸腔穿刺术是诊断恶性胸腔积液最常用的方法，但诊断率只有65%^[8]^，多次送检可以提高诊断率。内科胸腔镜以及电视胸腔镜手术（video-assisted thoracic surgery, VATS）在直视下观察胸膜情况，并能活检可疑的部位，敏感性达90%-100%^[2, 9]^，是所有检查中敏感性最高的诊断方法。但是内科胸腔镜和VATS均需要特殊的设备，并由专科医生操作，因此限制其在临床中常规应用。

PET和PET/CT主要通过摄取值的高低鉴别肺部良恶性肿瘤，与CT相比，PET和PET/CT诊断肺癌纵隔淋巴结转移的敏感性和特异性更高，能比常规的术前检查发现更多的远处转移，从而减少无效开胸率。PET也有助于鉴别胸腔积液的性质，其敏感性、特异性、阳性预测值和阴性预测值及准确率分别为95%-100%、67%-71%、63%-95%、67%-100%和80%-92%^[1, 10-14]^，高于胸水的细胞学检查，而与胸腔镜下胸膜活检的结果类似。本研究的结果显示如果仅以代谢值（胸膜SUVmax≥2.5）作为诊断标准，其诊断恶性胸腔积液的敏感性与胸腔穿刺术类似，只有59.3%，低于国外的研究，其主要原因可能与病例的选择有关，例如国外的研究选择CT胸膜异常的患者行PET检查，而本研究并没有专门选择胸膜增厚或多发结节的患者，因此如果患者胸膜转移病灶小，受PET空间分辨率的影响，PET容易出现假阴性。因此PET并非鉴别肺癌合并胸腔积液性质的有效诊断方法，特别是CT未显示胸膜有异常改变时。

PET/CT融合PET和CT技术，能准确地定位代谢异常的解剖部位，近年来已逐步取代PET用于肺部肿瘤的鉴别诊断和分期。Kim等^[1]^的研究显示PET/CT是鉴别胸腔积液性质的可靠方法，其中胸膜形态异常且代谢升高是诊断恶性胸腔积液的最佳参数，其敏感性为87.5%，特异性为88.8%，阳性预测值为95.2%，阴性预测值为72.7%。本研究的敏感性和特异性基本与Kim等^[1]^结果类似；本研究另一个重要结果是，PET/CT诊断肺癌合并恶性胸腔积液的假阳性率低，只有4.4%，而假阴性率高达50.0%，此结果与PET/CT诊断肺癌纵隔淋巴结转移的假阳性较高、假阴性低相反。因此，对于PET/CT阴性的胸腔积液，需胸腔穿刺术或胸膜活检术进行病理学确认，而PET/CT阳性的结果可信度更高，如果多次细胞学阴性，应该对PET/CT异常的胸膜行胸腔镜活检。

虽然PET/CT是敏感性、特异性和准确性较高的诊断肺癌合并恶性胸腔积液的无创检查，但存在较高的假阴性，其主要原因可能是当胸膜转移病灶较小时，受空间分辨率的影响而不能显示代谢异常。另外，本研究中有2例通过临床随访诊断良性胸腔积液，由于患者接受化疗，可能会影响对胸腔积液性质的判断，因而高估敏感性和特异性，但其例数少，而且不影响阳性预测值的结果。

总的来说，PET/CT作为无创检查，诊断肺癌恶性胸腔积液的敏感性和特异性较高，假阳性率低，对PET/CT阴性的胸腔积液需病理学检查确认。
